# A novel *Enterostomula* (Platyhelminthes, Prolecithophora) species from two brackish lakes in Japan

**DOI:** 10.3897/BDJ.8.e47161

**Published:** 2020-02-17

**Authors:** Nao Omi

**Affiliations:** 1 Unaffiliated, Tsukuba, Japan Unaffiliated Tsukuba Japan

**Keywords:** free-living, Pseudostomidae, Rhabditophora, Turbellaria

## Abstract

**Background:**

The genus *Enterostomula* Reisinger, 1926 belongs to the family Pseudostomidae and comprises generally small and often conspicuously coloured species living on hard bottoms, in gravel and amongst algae. The Pseudostomidae comprises approximately 44 known species from Europe as well as North and South America. Previously, only one species, *Allostoma durum*, had been recorded in Japan. Known *Enterostomula* species are predominantly found in marine and brackish habitats.

**New information:**

I collected seaweed and sand samples from two brackish lakes near the coast of Shimane Prefecture, Japan and isolated turbellarians from them. The animals were observed as both living and preserved. Here, I describe a novel *Enterostomula* species with two dorsal black bands and a thick bursal wall.

## Introduction

Diverse organisms inhabit the brackish waters of Lakes Shinji and Nakaumi which are situated on Japan’s main island. Amongst these, I found a novel species of *Enterostomula* in 2014. This is the first pseudostomid to be found in a Japanese brackish lake.

The genus *Enterostomula* Reisinger, 1926, belongs to the family Pseudostomidae Graff, 1904-08, which represents one of four families of the order Prolecithophora (Noren and Jondelius 1999, Jondelius U. et al. 2001, Tyler S et al. 2016, Tyler S et al. 2019). Prolecithophora is a group of small flatworms (generally < 1 cm in length), which are usually shaped like a spindle or in the body wall which often have purple, yellow, red and brown pigments (Karling 1978, Karling 1993, Noren and Jondelius 2002).

Characteristic features of *Enterostomula* species include a bursa, a vaginal pore and an unpaired spermatic duct (Reisinger 1926, Cannon 1986). To date, only three *Enterostomula* species from Europe, North America, South America and China have been described (Tyler S et al. 2016, Ma et al. 2014). In addition, of all Pseudostomidae species, only *Allostoma durum* (Fuhrmann 1896) was described from Japan (Westblad 1955). Although the ecology of the genus *Enterostomula* has not been extensively studied, they are considered free-living worms of marine and brackish environments.

## Materials and methods

Sand and seaweed samples were collected from the Sakai Channel, the Sea of Japan and Lakes Shinji and Nakaumi near the coast of Shimane Prefecture, Japan, in 2014. *Enterostomula* species were found only in the samples collected from Lakes Shinji and Nakaumi (salinity, 1‰–20‰) and not from the Sakai Channel or the Sea of Japan (salinity, > 27‰).

The two lakes are connected by the Ohashi River and Lake Nakaumi connects to the Sea of Japan through the Sakai Channel. Lake Shinji covers an area of 79.25 km^2^, with salinity levels ranging from 1‰ to 3‰, whereas Lake Nakaumi covers an area of 86.2 km^2^, with salinity levels ranging from 1‰ to 25‰. The stable brackish water system comprises outgoing fresh water from the river, combined with the incoming tidal flow of seawater from the Sea of Japan.

algae and sand samples were collected from depths ranging from 0.1 to 1 m. Specimens were sorted and removed from the substrates under a stereomicroscope. All specimens were first studied alive using light microscopy. Afterwards, they were fixed with 10% formalin and embedded in paraffin after anaesthetisation with an isotonic magnesium chloride solution. Sagittal and frontal serial sections (3–6 µm) were stained using Haematoxylin & Eosin or the Azan method (Romeis 1989). Sectioned specimens (NMST-Pl 6298–6300) were deposited at the National Museum of Nature and Science (Tokyo).

## Taxon treatments

### Enterostomula densissimabursa sp. n.

634F84CC-1A1D-5C86-8BEF-26F59C944E0B

urn:lsid:zoobank.org:act:D08DBB74-3713-4481-B0C9-1E474189DAAB

#### Materials

- **Type status:** Holotype. **Taxon:** scientificName: *Enterostomula densissimabursa* sp. nov.; kingdom: Animalia; phylum: Platyheminthes; order: Prolecithophora; family: Pseudostomidae; genus: Enterostomula; taxonRank: species; **Location:** waterBody: Shinji Lake; island: Honshu (Main island of Japan); country: Japan; countryCode: Japan/JP; stateProvince: Shimane; **Identification:** identifiedBy: Nao Omi; **Event:** year: 2014; month: 8; habitat: Sand and seaweed; **Record Level:** language: en; institutionID: NMST-Pl 6300 (sagittal serial section)
- **Type status:** Paratype. **Record Level:** scientificName: *Enterostomula densissimabursa* sp. nov.; institutionID: NMST-Pl 6298 (Frontal serial setion), NMST-Pl 6299 (Sagittal serial section)

#### Description

Body is short and cylindrical, 0.28–0.42 mm (average 0.34 mm, n = 12) long and 0.12–0.33 mm (average 0.17 mm, n = 12) wide. Anterior end is rounded; no ciliated grooves or pits; posterior end is sharpened slightly. Body colour is white; two black pigmented bands located at the dorsal anterior and opposite to end; posterior pigment band extends to the ventral side through the flank; the pigment lies just below the basement membrane of the epidermis (Fig. 1a, b, c). The basement membrane under the body surface is thick. The intestine is pale orange or white in the living specimen. The eyes, surrounded by black pigment, are located anteriorly and partially embedded in the brain. Each eye consists of a heart-shaped posterior part of two lenses and a single anterior lens (Fig. 2a). The brain has a weak tunic and is situated under the testis and posteriorly to the eyes; pigment granules can be seen on the brain surface (Fig. 2a). The testis is follicular, with a tunic near the anterior end. Two vasa deferentia from the testis pass lateral to the intestine. No distinct vesicula seminalis is present. The male copulatory organ, dorsal to the intesitine, is long and cylindrical, 80 µm × 20 µm, with thick-walls of circular and longitudinal muscles; vesicular granulorum is located anteriorly within the male copulatory organ and the penis is located internally within the copulatory organ and extends beyond the penis sheath (Fig. 2b). The penis sheath is flexible and thick. The ovary is unpaired, not fully separated from the vitellarium and lies dorsally to and posterior to the testis (Fig. 2c). The vitellarium is an unpaired structure extending dorsally from just behind the testes to the posterior part of the body. The bursa is long and large and is located on the posterior dorsal part of the animal. The posterior portion of the bursa is swollen with thick walls consisting of fibre bundles around a narrow lumen (Fig. 2b). The anterior portion of the bursa has a columnar structure. The bursa connects to a sclerotised portion of the spermatic duct. The rest of this duct has a swollen portion that opens through a sclerotic bursal mouthpiece (Fig. 2d, f). The vagina externa opens into the bursa and connects to the vaginal pore at the posterior end of the animal’s body (Fig. 2e). The pharynx is small and tube-shaped and is located near the posterior ventral end. The oesophagus is short. The common oral–genital opening is located near the posterior ventral end. The intestine with weak tunic lies posterior to the testis.

#### Etymology

The name of the new species refers to its thick-walled bursa.

#### Distribution

Lakes Shinji and Nakaumi near the coast of Shimane Prefecture, Japan

#### Taxon discussion

By having a common gonopore, a short droplet body shape, a tubular pharynx, a brain and intestine with a weak tunic and an ovary that is not fully separated from the vitellarium, *E. densissimabursa* sp. nov. belongs to the family Pseudostomidae. Its unpaired ovary, unpaired spermatic duct, bursa, vagina externa and unpaired vitellarium more specifically place this species in the genus *Enterostomula*. Amongst pseudostomids, both genera *Allostoma* and *Enterostomula* have a vaginal pore and a bursa; however, *Allostoma* has a paired spermatic duct, while *Enterostomula* has an unpaired spermatic duct (Reisinger 1926, Ruffin Jones Jr. 1941).

*Enterostomula densissimabursa* sp. nov. has two black pigment bands on its dorsal side. The pigment is typical in prolecithophorans. *Allostoma amoenum* Karling, 1962 and *Enterostomula graffi* (Beauckamp, 1913), also have pigmented bands (Beauchamp 1913, Ma et al. 2014, Karling 1962). In addition, although *E. graffi* is considered a marine species, it has been reported in the brackish waters of the Marmara Sea, some European seas,and the Atlantic (Ruffin Jones Jr. 1941, Ax 1959, Ax 2008).

*Enterostomula graffi* has two pairs of eyes, a short globular male copulatory organ and a thin-walled bursa, whereas *E. densissimabursa* sp. nov. has one pair of eyes, its male copulatory organ is long and cylindrical in form and its bursa is thick-walled (Ma et al. 2014, Ruffin Jones Jr. 1941, Beauchamp 1913).

*Allostoma amoenum* superficially resembles *E. densissimabursa* sp. nov. Both species have dorsal pigment bands, cylindrical male copulatory organs and a large bursa. In *A. amoenum*, the vesicula seminalis connects to the male copulatory organ; however, a distinct vesicula seminalis is not observed in *E. densissimabursa* sp. nov. The bursa of *A. amoenum* directly connects to the ovary without a spermatic duct; however, in *E. densissimabursa* sp. nov., the bursa connects to the ovary via the spermatic duct. The bursa of *A. amoenum* is a large cellular body, whereas the wall of the bursa of *E. densissimabursa* sp. nov. consists mainly of thick fibres of extracellular matrix and muscle bundles. Although *E. densissimabursa* sp. nov. has one distinct pair of black eyes, *A. amoenum* lacks eyes entirely (Karling 1962). In addition, while *A. amoenum* has a groove on the anterior body surface, *E. densissimabursa* sp. nov. does not have a distinct groove.

*Allostoma amoenum* is considered endemic to California, USA and is found in marine environments (Karling 1962). The new species, reported in the present study, inhabits brackish lakes at low salinities ranging between 1‰ and 20‰.

In addition to bursa and male copulatory organs, spermatic ducts were observed in 11 individuals amongst the 17 *E. densissimabursa* sp. nov. specimens collected. Additionally, the bursal mouthpiece, which is connected to a single ovary, was observed in only one specimen. The development of a bursal mouthpiece could be the final stage in the development of the genital system of the organisms.

*Enterostomula graffi* and *Allostoma catinosum* (Beklemischev, 1927) (see Beklemischev 1927) have been collected only from brackish waters, but are considered to stem from ancestors in the marine environment (Ruffin Jones Jr. 1941, Beauchamp 1913, Mack-Fira 1974, Ax 1959, Ax 2008), while *Allostoma pallidum* Beneden, 1861 (see Ax 2008) is a marine-euhaline immigrant, collected from both marine and brackish habitats. *Enterostomula densissimabursa* sp. nov. was collected from the brackish waters of Lakes Shinji and Nakaumi with salinities ranging between 1‰ and 20‰ and not from the high salinity (> 27‰) outlets of the Sea of Japan, suggesting that it is a genuine brackish water species, as Ax 2008 would call it. Nonetheless, I found at least seven other pseudostomid species in marine waters just outside the lakes, whereas only *E. densissimabursa* was found within the lakes. Therefore, *E. mursubursa* potentially occupies a niche to which other local pseudostomids have not adapted. To date, few studies have reported Pseudostomids in environments with such low salinities. Future studies on the genus should not only explore it in seawater environments but also in low-salinity environments.

## Supplementary Material

XML Treatment for Enterostomula densissimabursa

## Figures and Tables

**Figure 1a.**
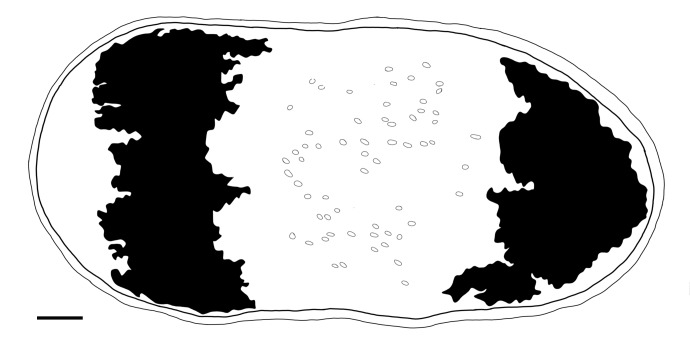
ventral view

**Figure 1b.**
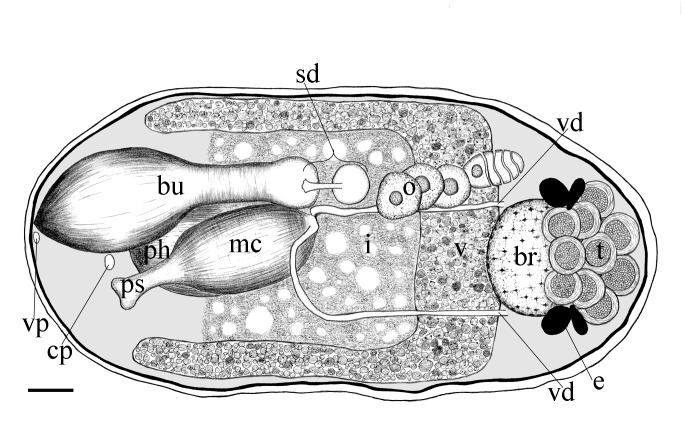
frontal reconstruction

**Figure 1c.**
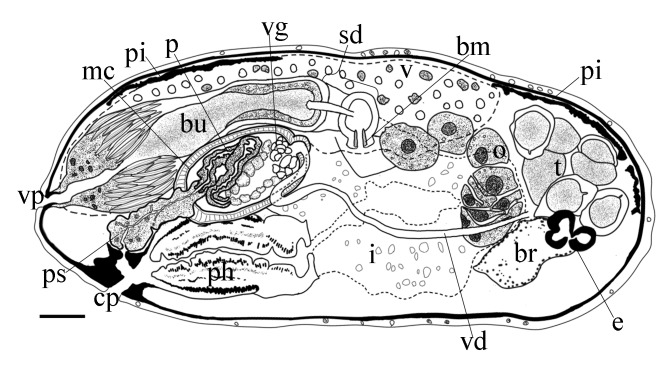
sagittal reconstruction

**Figure 2a.**
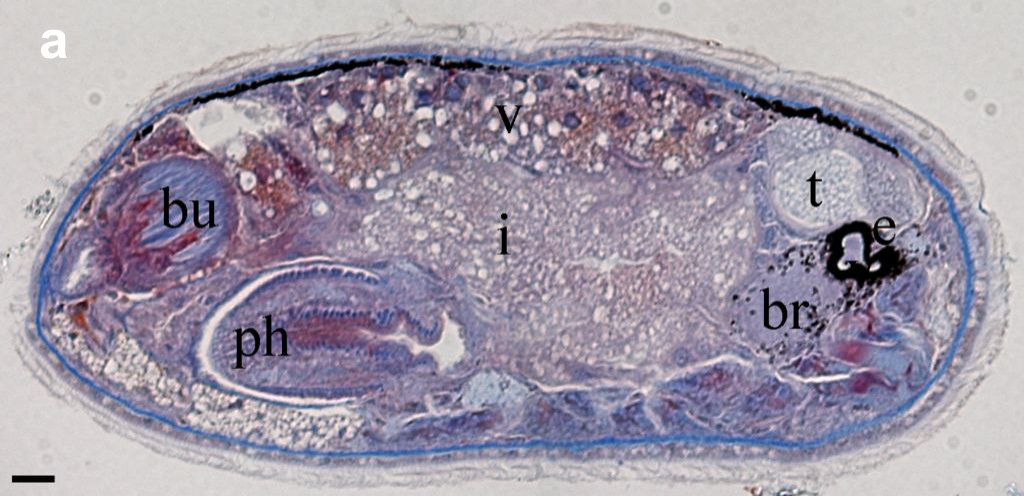
general view

**Figure 2b.**
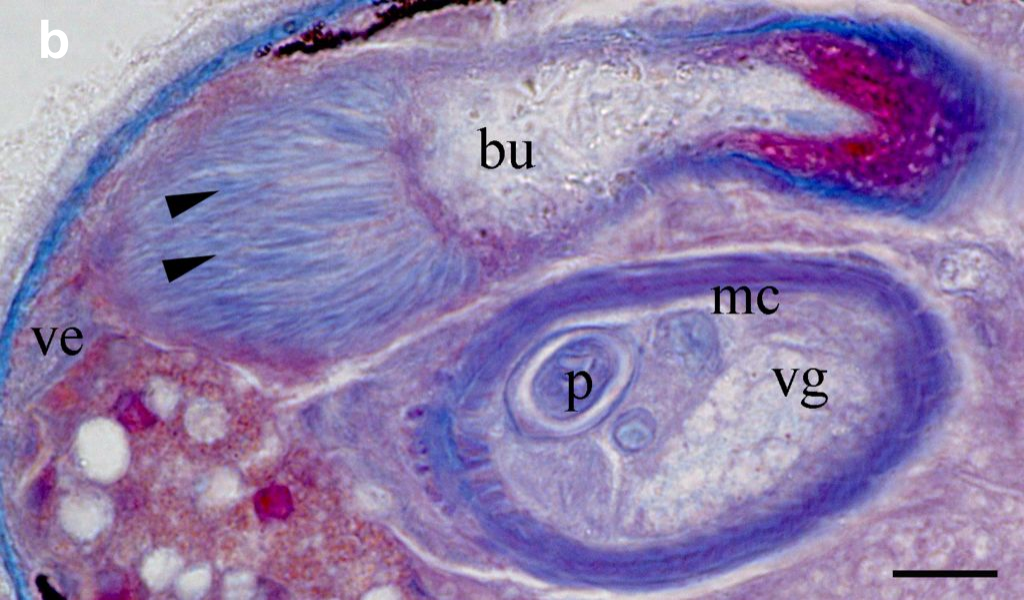
detail of the bursa and the male copulatory organ

**Figure 2c.**
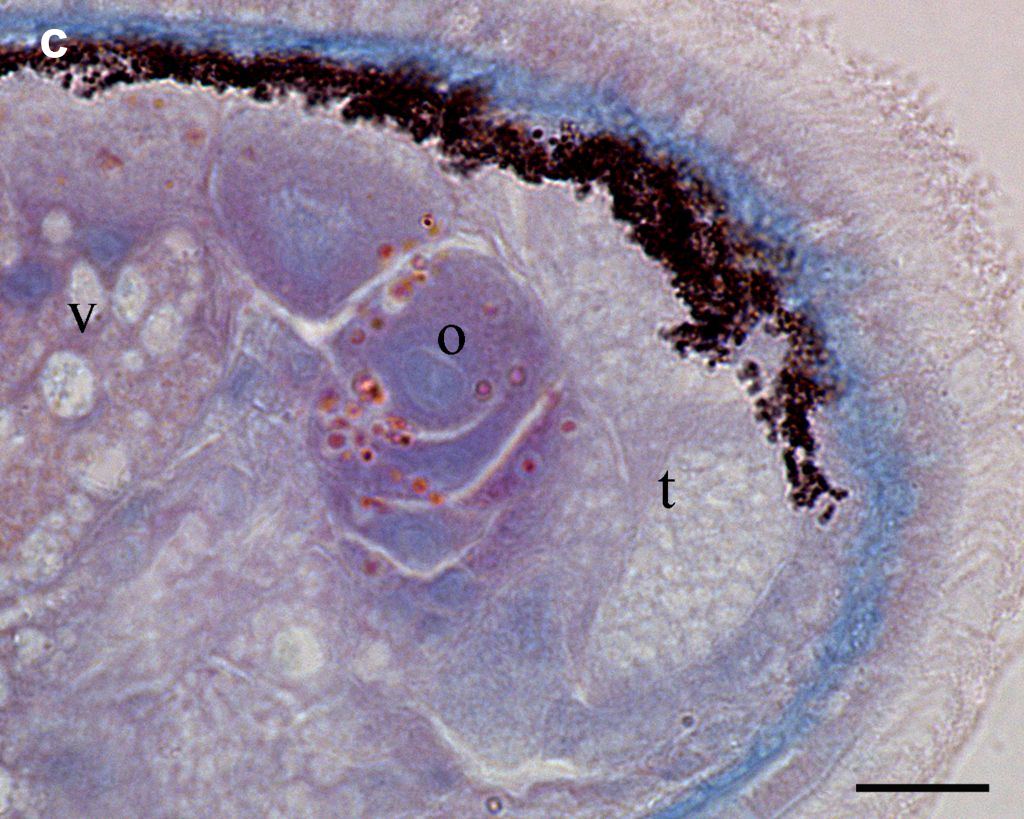
detail of the testis and ovary

**Figure 2d.**
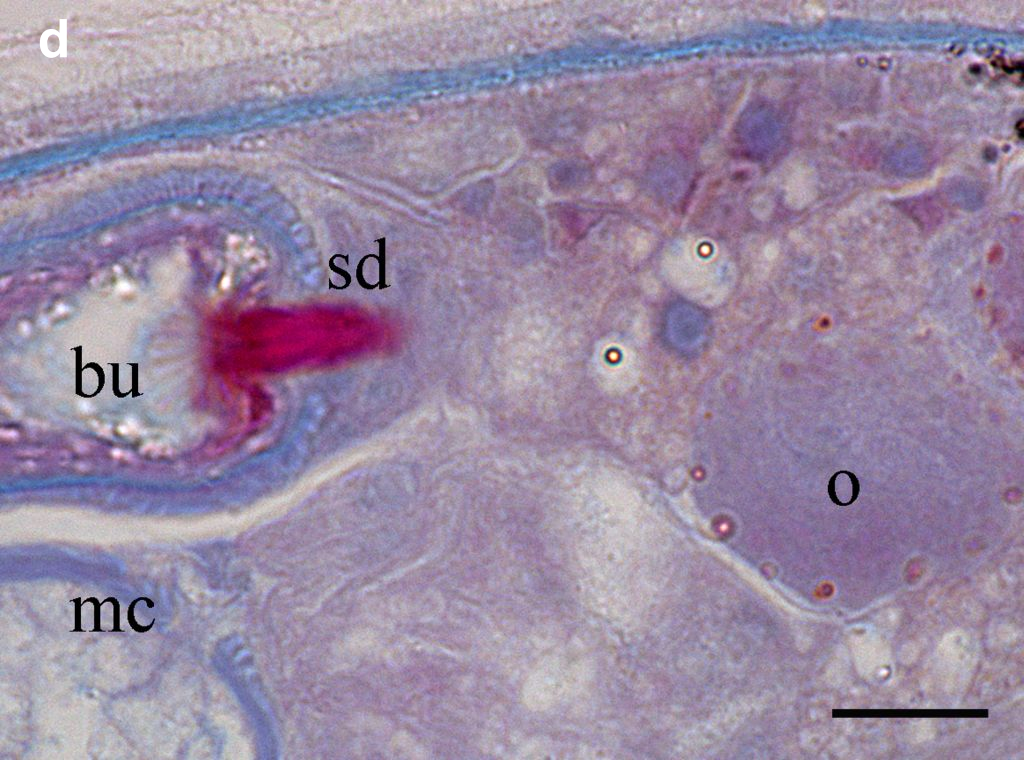
detail of the spermatic duct and the anterior end of the bursa

**Figure 2e.**
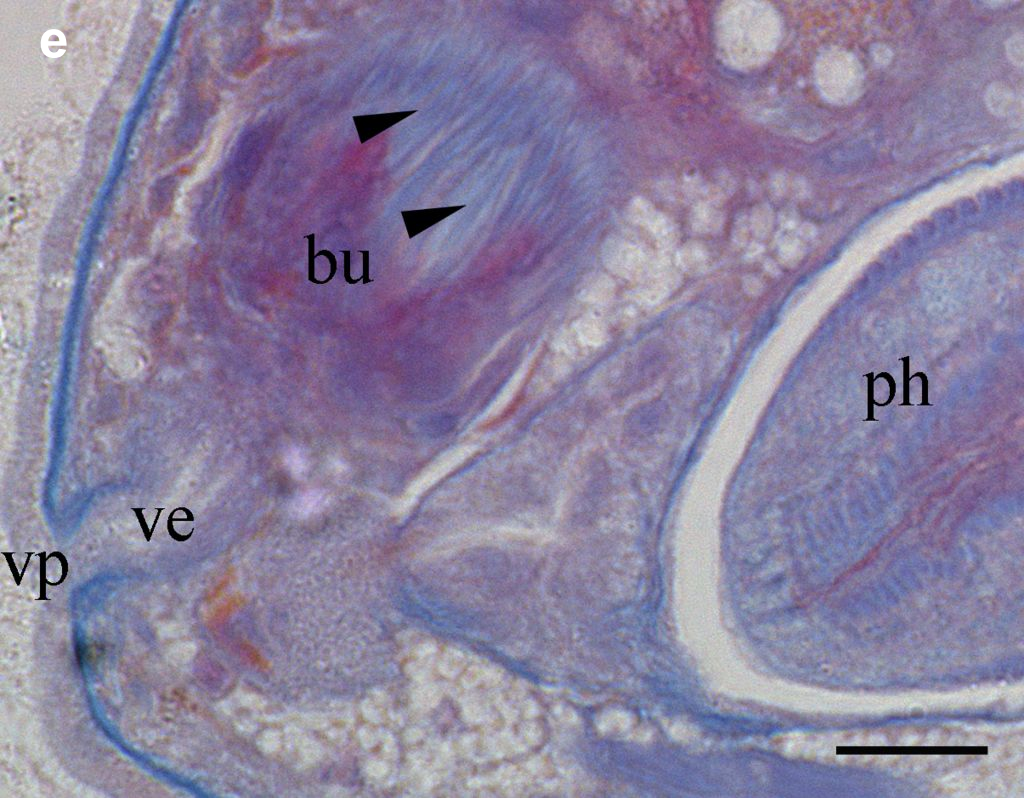
detail of the vaginal pore

**Figure 2f.**
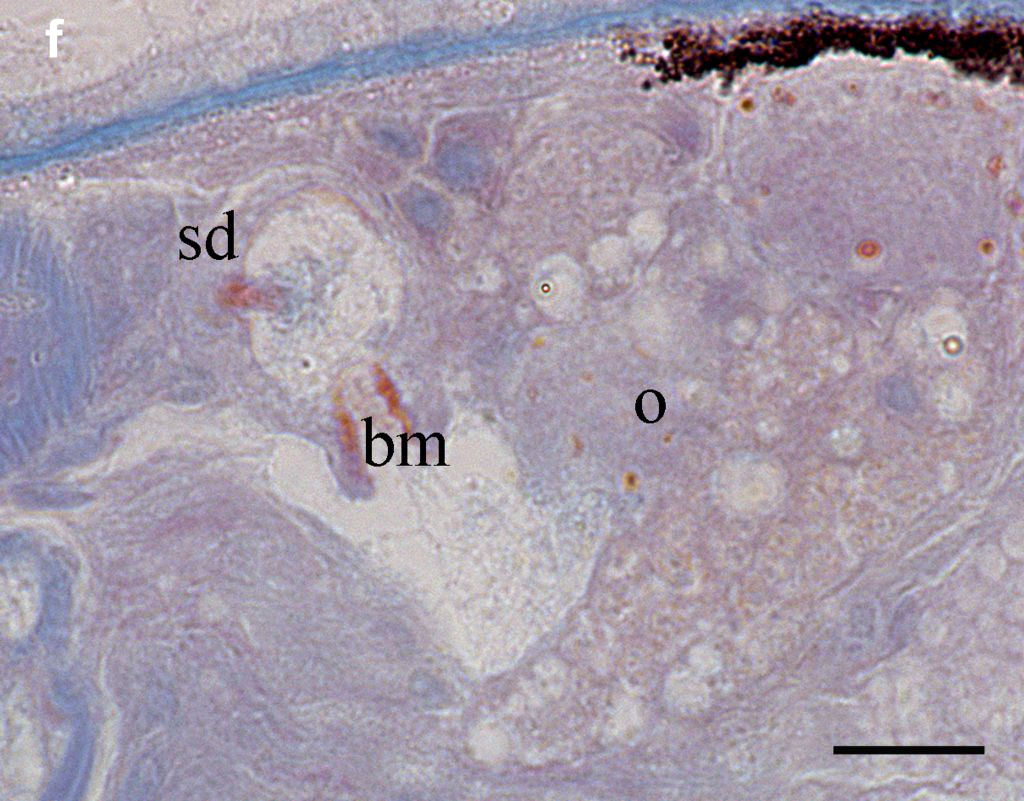
detail of the spermatic duct
